# Persistent Imbalance: Women’s Representation in North American Pediatric Cardiology Leadership Roles

**DOI:** 10.1016/j.jacadv.2025.101878

**Published:** 2025-07-23

**Authors:** Alaina K. Kipps, Audrey C. Marshall, Neha Bansal, Holly Bauser-Heaton, Nadine Choueiter, Devyani Chowdhury, Aarti Dalal, Molly Ehrlich, Lindsay R. Freud, Supriya S. Jain, Pei-Ni Jone, Kristin Laraja, Dalia Lopez-Colon, Sonal T. Owens, Christina Ronai, Ritu Sachdeva, Corey Stiver, Nicole Sutton, Terrie Vasilopoulos, Jennifer Co-Vu, American College of Cardiology Women in Cardiology-Adult Congenital Pediatric Cardiology Working Group

**Affiliations:** a Department of Pediatrics, Division of Cardiology, Stanford School of Medicine and Stanford Children’s Health Palo Alto, Palo Alto, California, USA; b Department of Paediatrics, Division of Cardiology, University of Toronto and Hospital for Sick Children Toronto, Toronto, Ontario, Canada; c Department of Pediatrics, Division of Pediatric Cardiology, Icahn School of Medicine at Mount Sinai and Kravis Children’s Hospital at Mount Sinai, New York, New York, USA; d Department of Pediatrics, Division of Pediatric Cardiology, Emory University School of Medicine and Children’s Healthcare of Atlanta, Atlanta, Georgia, USA; e Department of Pediatrics, Division of Cardiology, Sidney Kimmel Medical College of Thomas Jefferson University and Nemours Cardiac Center, Wilmington, Delaware, USA; f Department of Pediatrics, Division of Cardiology, Vanderbilt University School of Medicine, Monroe Carell Jr Children's Hospital, Nashville, Tennessee, USA; g Congenital Heart Center, University of Florida College of Medicine, Gainesville, Florida, USA; h Department of Pediatrics, Division of Pediatric Cardiology, New York Medical College, Maria Fareri Children’s Hospital at Westchester, New York, New York, USA; i Department of Pediatrics, Division of Pediatric Cardiology, Northwestern University Feinberg School of Medicine, Ann & Robert Lurie Children's Hospital, Chicago, Illinois, USA; j Division of Pediatric Cardiology, UMass Chan Medical School, Worcester, Massachusetts, USA; k Department of Pediatrics, Division of Pediatric Cardiology, University of Michigan Medical School, Ann Arbor, Michigan, USA; l Department of Cardiology, Boston Children’s Hospital, Department of Pediatrics, Harvard Medical School, Boston, Massachusetts, USA; m Department of Pediatrics, College of Medicine, The Ohio State University, Nationwide Children’s Hospital, Columbus, Ohio, USA; n Department of Pediatrics, Albert Einstein College of Medicine, Bronx, New York, USA; o Departments of Anesthesiology and Orthopaedic Surgery & Sports Medicine, University of Florida College of Medicine, Gainesville, Florida, USA

**Keywords:** gender, leadership, pediatric cardiology, subspecialty

## Abstract

**Background:**

Despite recent gender parity of physicians entering pediatric cardiology, representation of women leaders lags their male colleagues.

**Objectives:**

We sought to better understand the variation in women in leadership roles in pediatric cardiology.

**Methods:**

The gender of physicians in 16 prespecified leadership positions was collected by survey between July 2022 and January 2023 from pediatric cardiology programs with >5 cardiologists in North America. We analyzed the association of women leaders with center size (based on surgical volume), geographic region, presence of categorical fellowship program, and gender of division chief and department chair.

**Results:**

Across 99 centers, a median of 13 (Q1-Q3: 10-15) roles/center were identified. Women held 36.8% of all leadership roles and 35.1% of cardiology-specific roles. Only 13% of pediatric cardiology chiefs were women. Their programs had more women in subsection leadership roles than male-led centers (47% vs 36%, *P* = 0.028). A minority of leadership posts were shared among 2 physicians, yet more women than men shared their roles (5.4% women vs 2.5% men, *P* = 0.010). More men than women have dual leadership positions (15.1% men vs 9.9% women, *P* = 0.012). We found no association of center size, geographic region, presence of fellowship program, or gender of department chair with percent women leadership.

**Conclusions:**

Women hold fewer leadership positions across most subsections of pediatric cardiology programs, with more equitable distribution at centers led by women division chiefs. Women are more likely to share a leadership position with another cardiologist and less likely than men to hold more than 1 leadership post concurrently.

Medical institutions striving for inclusivity must critically examine structural imbalances in leadership roles. In the field of pediatric cardiology, gender imbalance persists and influences the dynamics of the specialty. In a landscape where for many years >70% of first-year categorical pediatric residents are women, there has been a concomitant increase in female representation among pediatric cardiology fellows, reaching 53% for the class of 2023.^1^ Steady growth of female graduates has led to gender equity of U.S. board-certified pediatric cardiologists: from 34% women in 2002 to 40% in 2012 and 49% in 2022.^2^ Despite this gender-balanced, board-certified workforce, representation in leadership positions remains skewed toward men.^3^^,^^4^ Recent studies have shed light on issues of gender-related disparities in subspecialty compensation and inequity of leadership roles for pediatric cardiology programs.^3^^,^^5^ While representation is equal among fellows and early-career faculty, the proportion of women dwindles with ascending seniority. Acquisition of clinical leadership roles and endowed chairs falls well behind that of male pediatric cardiologists.^3^

The Women in Cardiology Section of the American College of Cardiology workgroup on Adult Congenital and Pediatric Cardiology aims to “address the unique challenges that women in Pediatric and Adult Congenital Cardiology encounter while strengthening the pipeline by creating and supporting professional development, mentoring, and networking opportunities through the Women in Cardiology Section.”^6^ Members from 15 different programs formed a writing group in 2022. Our study sought to build upon the prior work^3^ with the inclusion of more centers and the inclusion of nonfellowship programs to map the current leadership landscape across the field and explore the gender allocation and variation in leadership for each pediatric cardiology subspecialty. Understanding leadership trends among each subspecialty leadership position and across the field may facilitate solution generation to promote an equitable working environment.

## Methods

A list of 16 subspecialty leadership positions within a comprehensive hospital-based pediatric cardiology practice was generated with consensus achieved across the 15 authors (Table 1). Network contacts were sent this list to obtain the names and genders of the individuals holding these predesignated leadership roles for each center. Surveys were administered by study authors via electronic mail, text messages, or telephone calls between July 2022 and January 2023. Cardiologists at each site were asked to note 1) whether the leadership position was a recognized titled role within the program, 2) the name and gender of the individual in that position, and 3) whether any roles were held by more than 1 person (ie, “shared roles”). From collected leader names, we determined whether program individuals held multiple leadership roles (ie, “dual roles”). As no further information was sought about these physicians, it fell outside of human subject research and is institutional review board exempt.

**Table 1:** Defined Leadership Roles and Distribution to Women in 99 North American Heart Centers

	Total Leadership	Total Women	% Women
Hospital-level/Department of Pediatrics Leadership Roles			
Pediatric department chair	98	36	36.7
Hospital CEO	97	24	24.7
Pediatric Residency Director	96	67	69.8
Cardiology Division Leadership Roles			
Cardiology Division Chief	100^a^	13	13.0
Cardiac Intensive Care Unit Medical director	94	31	33.0
Acute Care Cardiology Unit Medical director	60	25	41.7
Outpatient Cardiology director	64	30	46.9
Echocardiography lab director	92	37	40.2
Fetal imaging director	87	57	65.5
cMRI/Advanced cardiovascular imaging director	74	25	33.8
Adult Congenital Heart Disease director	95	31	32.6
Pulmonary hypertension director	68	24	35.3
Transplant/heart failure director	70	24	34.3
Catheterization laboratory director	98	17	17.3
Electrophysiology director	90	18	20
Exercise laboratory director	62	20	32.3
Preventive cardiology director	43	23	53.5
Research director	46	20	43.5
Fellowship Program Director	70	31	44.3

cMRI = cardiac magnetic resonance imaging; CEO = chief executive officer.

a There are programs with shared leadership roles (eg, 3 sites have 2 division chiefs and 2 programs without a named division chief); see Table 2.

To be more comprehensive, we sought to include a variety of practice types, not isolated to academic programs with fellowship training. We recognized, however, that designated subspecialty leadership roles would be fewer and likely more informal in very small practices. We started with all Society of Thoracic Surgeons (STS) pediatric cardiology centers in 2022 and excluded practices with fewer than 5 identified staff cardiologists. We collected data on pediatric cardiology leadership roles, including the division chief and each subspecialty’s directorship. Additionally, we queried institutional leadership, including the hospital’s Chief Executive Officer, the Chair of Pediatrics, and the Residency Training Program Director. As a proxy for practice size, we used reported STS surgical volumes and classified programs into 3 groups: small with <150 cases/year, medium with 150 to 350 cases/year, and large with >350 cases/year.^7^ We noted the broad geographic region in which the program was based and the presence of a categorical pediatric cardiology fellowship program.

Categorical measures were summarized as counts and percentages, and continuous measures as medians. Rates of women in leadership roles were summarized as percentage with exact binomial 95% CIs. A scaled Poisson regression to model for the rate of women in all leadership roles accounted for varying numbers of leadership roles per center. First, separate models were fit to compare rates of overall female leadership roles by center size, geographic location, presence of fellowship, division chief gender, and department chair gender. Then, a multivariable model including factors was run; effects were quantified with rate ratios and 95% CIs. For shared and dual roles, the proportion of women and men in those roles was compared to the proportion of each gender in overall leadership roles using Z-tests for 2 proportions. The association between dual roles and site size was evaluated with chi-square tests, and group differences in total leadership roles between programs with and without dual roles were assessed with the Mann-Whitney test. Analyses were conducted in JMP Pro 17 (SAS Institute Inc).

## Results

Of 121 STS pediatric cardiology centers in June 2022, 106 met inclusion criteria. Complete data were gathered on 99 centers (93% completion rate). A median of 13 (Q1-Q3: 10-15) roles/center were identified, with 4 (Q1-Q3: 3-6) held by women. Across all roles, including the institutional ones, women held 36.8% of leadership roles. Nearly all programs had a department of pediatrics chair, of which 37% were held by women. Women chief executive officers were less common at 25%, while it was more common to have a woman lead the pediatric residency program at 70%. The mean percent of women in cardiology-specific leadership roles was 33%, ranging from 0% to 85%. A quarter of programs had ≥50% of their cardiology leadership positions held by women, while 4 centers had no women leaders.

Over 85 programs had a named leader in cardiac intensive care, echocardiography, adult congenital heart disease, cardiac catheterization, and electrophysiology (Table 1). Less common roles were preventative cardiology (43) and research program directorships (46). The lowest representation of women among all roles was for pediatric cardiology chiefs: 13% of programs were led by women (Table 1). There was considerable variability across subspecialty sections: the highest proportion of female leadership was in fetal echocardiography (66%), while only 17% of interventional cardiology leaders and 20% of electrophysiology leaders were women (Central Illustration). A near-even distribution was found for ambulatory (47% women) and preventive cardiology (54% women) sections.

**Central Illustration:**
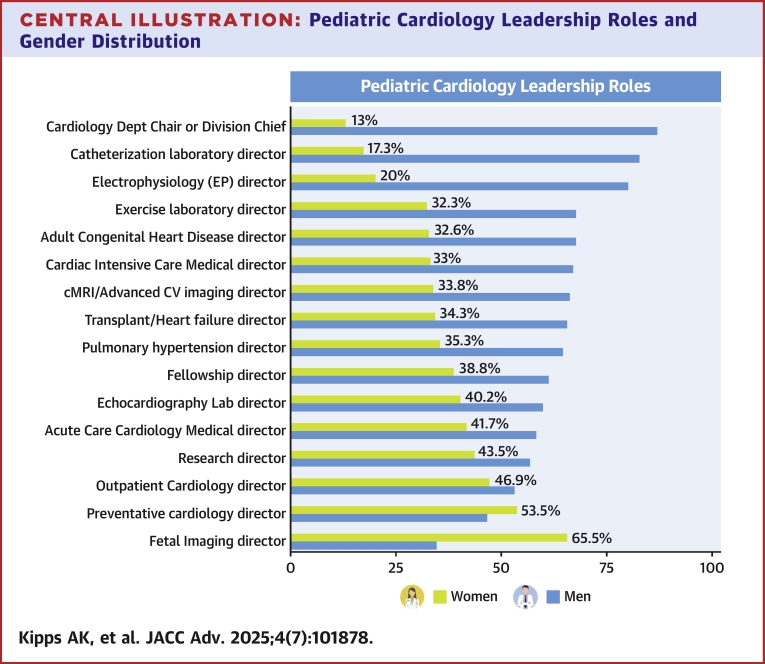
Pediatric Cardiology Leadership Roles and Gender Distribution The variation in women’s representation among all major pediatric cardiology clinical subsection and programmatic roles is illustrated. There is underrepresentation of women in leadership roles across most areas, with the lowest among chiefs, interventional cardiology, and electrophysiology. The highest representation is in the areas of fetal cardiology and preventive cardiology. cMRI = cardiac magnetic resonance imaging; CV = cardiovascular.

The only site characteristic significantly associated with the percentage of women in leadership roles was the presence of a female pediatric cardiology chief. Cardiac programs led by women had more women in subsection leadership roles (47% vs 36%, *P* = 0.028, Table 2). This association between the presence of a female pediatric cardiology chief and the percentage of women in leadership roles remained in multivariable analysis (rate ratio = 1.23, 95% CI: 1.08-1.42, *P* = 0.003). Among the 24 programs with ≥50% female cardiology leadership, 8 had a woman division chief. In the United States, the Northeast trended toward a higher percentage of women leaders (43%) than the West (32%), South (31%), or Midwest (34%) (*P* = 0.133). Program size, based on annual surgical volume, fellowship program, or department chair gender, was not significantly associated with leadership distribution (Table 2). Examining specific subsections (Supplemental Tables 1 and 2), there were fewer female cardiac magnetic resonance imaging leaders in medium- (23%) or high-volume (17%) centers compared to low-volume centers (39%, *P* = 0.015). Additionally, there were more women fetal imaging directors in centers with fellowship programs (72%) compared to those without fellowships (48%, *P* = 0.048).

**Table 2:** Percentage of Females in Pediatric Cardiology Leadership Roles by Center Characteristic

Center Characteristic	N	Rate (%) of Women Leaders^a^ (95% CI)	Rate Ratio (95% CI)	Overall *P* Value
Overall	99	35.1 (32.4, 37.9)	--	
Size (cases/y)				0.857
Small (<150)	39	34.9 (30.3, 39.7)	0.98 (0.85, 1.11)	
Medium (150-350)	36	34.1 (29.7, 38.6)	0.98 (0.86, 1.13)	
Large (>350)	20	36.0 (30.1, 418)	Ref	
Region (those in the United States)				0.133
Midwest	23	33.6 (28.2, 39.3)	0.97 (0.81, 1.14)	
South	37	31.8 (27.5, 36.4)	0.91 (0.78, 1.06)	
West	18	32.4 (26.1, 39.2)	0.92 (0.75, 1.12)	
Northeast	17	43.0 (36.3, 49.9)	Ref	

	**N**	**Rate (%) of Women Leaders**^b^**(95% CI)**	**Rate Ratio (95% CI)**	**Overall *P* Value**

Presence of fellowship program				0.147
Yes	67	38.3 (35.0, 41.7)	1.09 (0.97, 1.23)	
No	32	33.5 (27.8, 39.4)	Ref	
Gender of Division Chief				0.028
Female	13	47.2 (39.2, 55.2)	1.16 (1.02, 1.31)	
Male	87	35.6 (32.5, 38.7)	Ref	
Gender of Department Chair				0.785
Female	36	37.7 (32.9, 42.6)	1.01 (0.92, 1.12)	
Male	61	36.6 (33.0, 40.3)	Ref	

CEO = chief executive officer.

a Excluding CEO, Department Chair, and Pediatric Residency Program Director.

b Further excluding Pediatric Cardiology Division Chief.

Of the 30 shared cardiology roles, 10 were pairs of 2 women, 7 were of 2 men, and 13 were women partnered with men (Table 3). Shared roles for women accounted for a significantly higher percentage of total roles than male shared roles: 5.4% vs 2.5% (*P* = 0.010). Compared to the proportion of total leadership roles, women were overrepresented in shared roles (77% of shared vs 35% of overall roles, *P* < 0.001). The proportion of shared roles varied across subsections from 0% to 19%. Adult congenital heart disease (19%), pediatric heart failure (14%), and cardiovascular intensive care unit (CVICU) (11%) had the most shared posts, while outpatient, electrophysiology, and research programs had no shared positions.

**Table 3:** Shared Roles and Dual Roles and Gender Distribution in Pediatric Heart Centers

	Total Leadership Roles	Leadership Roles: Women (%)	Leadership Roles: Men (%)	Comparison of the % of Women in Shared or Dual Roles vs % of Total Cardiology Roles Occupied by Women^a^	*P* Value
All cardiology roles	1,213	426 (35%)	787 (65%)	-	
Shared roles	30	23 (77%)	20 (67%)	77% shared vs 35% across all roles	*P* <0.001
Woman and woman	10	10	--		
Man and man	7	--	7		
Woman and man	13	13	13		
Dual roles	161	41 (25%)	120 (75%)	25% dual vs 35% all roles	0.023

a *P* values from z-test for 2 proportions.

Women held 25% of 161 dual roles (Table 3). Women leaders with 2 or more titles comprise 9.9% of their total roles, compared to men with 2 or more titles, which accounted for 15.1% of their total leadership (*P* = 0.012). Women are less likely than men to hold additional titles: compared to the total leadership distribution, women were underrepresented in dual roles (25% dual and 35% overall, *P* = 0.023). In contrast, men who hold 1 leadership post are more likely to hold a second directorship (75% dual and 65% overall, *P* = 0.015). Cardiology division chiefs who held another section leadership position were encountered in 42% of centers, accounting for 26% of all dual roles. The most common combinations for division chiefs were to lead programs in catheterization (8), CVICU (6), echocardiography (5), outpatient (4), or electrophysiology (4). More male division chiefs (n = 39 or 45% of male chiefs) held another post than female division chiefs (n = 3 or 23% of female chiefs). The existence of dual roles for division chiefs was associated with size of the program (*P* = 0.003) and the number of named positions (*P* = 0.011). Division chiefs were more likely to have dual roles at smaller programs (62%) compared to medium (28%) and large (10%) centers.

## Discussion

Our cross-sectional study of 99 North American pediatric cardiology programs found that women are underrepresented in leadership, particularly among senior leadership positions (division chiefs) and in the procedural subspecialties, electrophysiology and interventional cardiology. A prior survey of cardiac programs participating in fellowship training documented a similar distribution for chiefs,^3^ while more recently one documented further skew toward male representation for this role.^4^ Our findings build on these earlier analyses^3^^,^^4^ by including nonfellowship programs, analyzing additional subspecialty leadership roles, and introducing the novel concepts of role-sharing and dual leadership. There was considerable variation in the representation of women in leadership roles across the subspecialties, with the highest representation in the areas of fetal, ambulatory, and preventive cardiology.

The classic metaphorical explanations for lower female leadership in many fields, “leaky pipeline” (the loss of women faculty along the path to leadership) and the “glass ceiling” (denoting an invisible barrier to advancement),^8^^,^^9^ fail to address the multiplicity of challenges that women face in pursuit of leadership.^10^ One of the most significant barriers for women seeking leadership roles is persistent gender bias, where men are seen as more competent and capable of leadership than women, even with equivalent credentials.^11^^,^^12^ Some believe this issue will self-resolve and women will gain equality in leadership representation as more women physicians join each field. However, several studies indicate a persistent lag in promotion rates.^13^^,^^14^

Factors influencing leadership appointments, including academic productivity, clinical expertise, and demonstration of leadership skills, are often informal, lacking transparency or standardization. Broadly shared conscious and unconscious psychological associations about women, men, and leaders impact the selection process. Often, women leaders encounter a dilemma: if they are highly communal, they may be criticized for not being agentic enough. Women who are highly agentic may be criticized for lacking communion. Additionally, self-promotion is not communal and can feel hazardous and/or unnatural for women.^10^ We believe division chiefs determine sectional leadership, but no data exist regarding how these roles are filled or their rate of turnover. Lack of transparency and oversight can result in favoritism and noncompetitive long-term sectional appointments.

We found more equitable distribution at centers led by women division chiefs; several had ≥50% of subsectional leadership roles held by women. Division chiefs typically appoint subsection leadership posts. Female cardiology chiefs may be more likely to actively recruit and train women faculty to lead sections or be more intentionally equitable in the distribution of leadership roles. This could be driven by a desire for more diversity or relate to homophily: in the presence of women leaders, more women can advance their leadership goals and overcome governance tokenism.^15^^,^^16^ A woman division chief may also temper the influence of gender bias while stimulating other women faculty to envision their own capacity for leadership. Empirical evidence in several fields suggests that when women rise to influential positions, they are more likely than men to support other women.^17^^,^^18^ High-ranking women can help legitimize other women’s contributions and temper gender stereotypes.^19^ The correlation of women chiefs with more equitable subsectional leadership could reflect an inclusive programmatic culture established by prior chiefs (who were usually male) with succession by a woman who continued to build gender-equitable leadership teams.

Within pediatric cardiology, “gender tracking” may contribute to the uneven distribution, with greater representation of women in the communal, noninvasive areas of cardiology compared with the underrepresentation of women in more highly compensated procedural and agentic areas of interventional cardiology and electrophysiology.^20^ The well-represented areas may offer greater schedule flexibility or foster better work-life integration. The leaky “applicant pipeline” problem has been used to explain the low numbers of women in adult interventional cardiology and electrophysiology.^21^^,^^22^ Concerns about radiation, lack of female role models, a perceived “old boys’ club” culture, and discrimination/harassment concerns deter women from these procedural specialties.^22^ According to the 2023 Doximity Physician Compensation Report, women pediatric cardiologists earn 9.2% less than men. This gap compounds significantly over a career and translates to over a million dollars in lost wages.^23^

A novel finding is a greater likelihood of women in pediatric cardiology to share a leadership position,^24^ while men have a greater likelihood to hold more than 1 leadership post concurrently. The dominance of male division leaders holding another sectional title suggests a possible intervention: these chiefs could recruit another faculty, perhaps a qualified woman (especially in programs with female leader underrepresentation), to assume their subspecialty leadership role(s). Even in smaller volume centers where more male chiefs hold dual roles, broader leadership distribution should be feasible. Medicine traditionally promotes the idea of unitary command and has seldom embraced the collaborative practice of shared leadership.^25^ On one hand, having a woman join a shared post previously led by a man alone could bolster women in leadership. However, chiefs need to be mindful of the gender distribution of shared subsectional leadership: sharing posts may diminish a leader’s influence and status compared to being a singular leader.

We cannot directly determine whether individual centers have disproportionate leadership without an accurate count and gender distribution for each practice and subspecialty. Workforce distribution data are lacking among the pediatric cardiology subspecialties, but we can extrapolate from recent annual certification enrollment surveys (2018-2022),^26^ which indicate subspecialties have uneven gender distribution. More men primarily identified as a pediatric interventional cardiologist (81% of 44 respondents) or electrophysiologist (79% of 42 respondents). These are similar to the proportions of men leading these sections: 83% in interventional cardiology and 80% in electrophysiology. Similarly, more women identify fetal cardiology as their primary field (81% of 16 respondents), congruent with the 66% female leadership among fetal directors. However, this pipeline explanation is less helpful for other major subspecialties. A more even distribution of genders was seen among the 90 respondents identifying as noninvasive imaging cardiologists (50% female), 54 CVICU specialists (52% female), and 25 heart failure/transplantation specialists (52% female). These even distributions are less consistent with women’s leadership in echocardiography, CVICU, or heart failure/transplantation sections (40%, 33%, or 34%, respectively).

### Study Limitations

Several study limitations must be considered. The STS center list may have missed cardiologist groups that outsource cardiothoracic surgery. The high number of subspecialty leadership positions reported by programs may reflect how we solicited information in table form. This has the potential for “over-reporting” leadership roles and may impact the share of women in each role. We used “gender” to refer to how individuals present themselves professionally, which may not necessarily reflect their personal gender identity. We did not explicitly collect data on gender identity from each individual and relied on a single cardiologist from each site to note the names and genders of each leader. As such, our categorization may not fully capture the diversity of gender experiences in pediatric cardiology leadership. We did not query the total number of cardiologists at each center since this is often in flux, and partial or dual appointments add complexity to achieving an accurate count. We did not inquire about medical school leadership roles or directorships of collaboratives, societies, or research networks. The level of compensation or amount of protected time, if any, that cardiologists leading the subsections receive was also out of scope. Among the writing group, many hold leadership positions, with great variety in workload and compensation (both in time and pay) for leadership.

Our next study was to better understand the ascent of the minority group of women division chiefs to leadership. Using qualitative thematic analysis, we sought to better understand what factors influenced their careers, which gender bias patterns they encountered, and their ideas on effective strategies to address the persistent gender leadership gap in our field. Additionally, our committee helped prepare an American College of Cardiology-sponsored national survey, which will provide an additional opportunity to study gender parity in areas of compensation, academic rank, and subspecialty choice among pediatric cardiologists.

## Conclusions

We found that women hold fewer leadership positions across most subsections of pediatric cardiology programs, with more equitable distribution at centers led by women division chiefs. Additionally, novel findings include that women are more likely to share a leadership position and less likely than men to hold more than 1 leadership post concurrently. Institutions should drive policies to improve this imbalance, focusing on sponsorship, transparent selection processes, and support for all faculty with household caregiving responsibilities. Promoting equity in leadership strengthens institutional diversity and can enhance innovation and clinical outcomes.

Perspectives **COMPETENCY IN PROFESSIONALISM:** Subsection and division leaders can influence the research agenda and allotment of time and resources to their faculty. The discrepancies between the gender distribution of fellows and board-eligible pediatric cardiologists and programmatic leadership of pediatric heart centers could have consequences on research agendas and quality improvement project prioritization.

**TRANSLATIONAL OUTLOOK:** The lack of equality in leadership roles hinders the diversity of perspectives that can provide innovative solutions to challenges in the clinical and research realms of our specialty.

## Funding support and author disclosures

The authors have reported that they have no relationships relevant to the contents of this paper to disclose.
