# Supplementary material for: Persistent Imbalance: Women’s Representation in North American Pediatric Cardiology Leadership Roles
Source: JACC Adv. 2025 Jul 23;4(7):101878. doi: 10.1016/j.jacadv.2025.101878 (PMC12418463; doi:10.1016/j.jacadv.2025.101878)
Supplement: Supplemental Material [file mmc1.docx]

Supplemental Material

**Table 1: Percent women in leadership roles for difference positions, stratified by program size**

**Table 2: Percent women in leadership roles for difference positions, stratified by presence of pediatric cardiology fellowship program**

**Supplemental Table 1: Percent women in leadership roles for difference positions, stratified by program size**

|  | **Small** | **Medium** | **Large** | **p-value^a^** |
| --- | --- | --- | --- | --- |
| **Hospital level/Department of Pediatrics Leadership Roles** | | | | |
| **Pediatric department chair** | 14/38 (37%) | 11/36 (31%) | 8/20 (40%) | 0.726 |
| **Hospital CEO** | 8/38 (21%) | 9/34 (26%) | 6/21 (29%) | 0.824 |
| **Pediatric Residency Director** | 24/35 (69%) | 25/36 (69%) | 15/21 (71%) | 1.0 |
| **Cardiology Department/Division Leadership Roles** | | | | |
| **Cardiology department or division chief/chair** | 4/41 (10%) | 7/35 (20%) | 2/20 (10%) | 0.401 |
| **Cardiac Intensive Care director** | 7/32 (22%) | 16/37 (43%) | 8/22 (36%) | 0.178 |
| **Acute care cardiology director** | 5/16 (31%) | 11/23 (48%) | 8/18 (44%) | 0.595 |
| **Outpatient cardiology director** | 9/20 (45%) | 14/25 (56%) | 6/18 (33%) | 0.351 |
| **Echocardiography lab director** | 14/36 (39%) | 13/31 (42%) | 6/20 (30%) | 0.692 |
| **Fetal imaging director** | 21/33 (66%) | 18/29 (62%) | 12/19 (63%) | 0.574 |
| **cMRI/Advanced CV imaging director** | 15/28 (54%) | 6/26 (23%) | 3/18 (17%) | **0.015** |
| **ACHD director** | 12/33 (36%) | 8/36 (22%) | 8/20 (40%) | 0.302 |
| **Pulmonary hypertension director** | 8/20 (40%) | 6/27 (22%) | 10/18 (56%) | 0.069 |
| **Transplant/Heart failure director** | 7/17 (41%) | 6/28 (21%) | 8/22 (36%) | 0.352 |
| **Catheterization laboratory director** | 7/37 (19%) | 5/35 (20%) | 5/22 (23%) | 0.700 |
| **Electrophysiology director** | 9/33 (27%) | 6/34 (18%) | 3/20 (15%) | 0.138 |
| **Exercise laboratory director** | 7/20 (35%) | 6/26 (23%) | 6/15 (40%) | 0.468 |
| **Preventative cardiology director** | 5/18 (28%) | 10/26 (39%) | 7/19 (37%) | 0.795 |
| **Research director** | 4/12 (33%) | 11/33 (33%) | 5/18 (28%) | 1.0 |
| **Fellowship director** | 6/16 (38%) | 12/30 (52%) | 9/20 (45%) | 0.509 |

Note: denominators represent total named positions for each category

a) Fishers exact test

**Supplemental Table 2: Percent women in leadership roles for difference positions, stratified by presence of pediatric cardiology fellowship program**

|  | **With fellowship programs** | **Without fellowship programs** | **p-value^a^** |
| --- | --- | --- | --- |
| **Hospital level/Department of Pediatrics Leadership Roles** | | | |
| **Pediatric department chair** | 25/68 (37%) | 11/30 (37%) | 1.0 |
| **Hospital CEO** | 20/66 (30%) | 4/31 (13%) | 0.080 |
| **Pediatric Residency Director** | 47/70 (67%) | 20/26 (77%) | 0.456 |
| **Cardiology Department/Division Leadership Roles** | | | |
| **Cardiology department or division chief/chair** | 10/68 (15%) | 3/32 (9%) | 0.514 |
| **Cardiac Intensive Care Medical director** | 21/65 (32%) | 10/29 (34%) | 1.0 |
| **Acute Care Cardiology Medical director** | 21/47 (45%) | 3/12 (25%) | 0.326 |
| **Outpatient Cardiology director** | 21/46 (46%) | 9/18 (50%) | 0.787 |
| **Echocardiography lab director** | 26/64 (41%) | 10/27 (37%) | 0.817 |
| **Fetal imaging director** | 43/60 (72%) | 12/25 (48%) | **0.048** |
| **cMRI/Advanced CV imaging director** | 15/52 (29%) | 10/22 (45%) | 0.188 |
| **Adult Congenital Heart Disease director** | 24/70 (34%) | 6/24 (25%) | 0.457 |
| **Pulmonary hypertension director** | 21/54 (39%) | 3/14 (21%) | 0.378 |
| **Transplant/Heart failure director** | 21/56 (38%) | 3/12 (25%) | 0.518 |
| **Catheterization laboratory director** | 12/68 (18%) | 5/30 (17%) | 1.0 |
| **Electrophysiology director** | 11/64 (17%) | 7/26 (27%) | 0.382 |
| **Exercise laboratory director** | 13/44 (30%) | 7/18 (39%) | 0.793 |
| **Preventative cardiology director** | 19/51 (37%) | 3/12 (25%) | 0.516 |
| **Research director** | 17/57 (30%) | 3/9 (33%) | 0.372 |

Note: denominators represent total named positions for each category

a) Fishers exact test
